# Supplementary material for: The modulatory properties of Astragalus membranaceus treatment on endometrial cancer: an integrated pharmacological method
Source: PeerJ. 2021 Aug 24;9:e11995. doi: 10.7717/peerj.11995 (PMC8395571; doi:10.7717/peerj.11995)
Supplement: Supplemental Information 1 [file peerj-09-11995-s001.docx]

Table S1: All 268 EC-related DEGs with high reliability

| id |
| --- |
| PTPRR |
| CA11 |
| FOXM1 |
| CDK1 |
| PCCB |
| MAD2L1 |
| OLFML2A |
| DCX |
| KIF4A |
| TYMS |
| SNCA |
| EPB41L3 |
| HN1 |
| CCNA2 |
| CKS2 |
| RPS6KA2 |
| HMBS |
| SLC25A15 |
| ANXA2 |
| CSDC2 |
| LMNB1 |
| TCEAL2 |
| TACC1 |
| CCNB2 |
| NFE2L3 |
| ENPEP |
| FAM184A |
| SYNE1 |
| CRIP1 |
| MT1F |
| CLIP3 |
| MAF |
| ZNF135 |
| DCHS1 |
| PKP3 |
| SDF2L1 |
| NEK2 |
| AP1S1 |
| ESPL1 |
| NEDD4 |
| BORA |
| CHEK1 |
| APBB1 |
| ARL4C |
| LDHA |
| SCD |
| PRR15L |
| SFN |
| NMT2 |
| ISG15 |
| MAGEH1 |
| UBE2S |
| CLDN5 |
| LGMN |
| NUDT11 |
| ORMDL2 |
| FGF2 |
| RRM2 |
| LRRC59 |
| GPRASP1 |
| MAGEL2 |
| SLC29A2 |
| CIRBP |
| MT1H |
| SNED1 |
| CDKN3 |
| PGD |
| ITPR3 |
| NAALAD2 |
| GOT1 |
| KPNA2 |
| BIRC5 |
| GHR |
| STARD13 |
| RACGAP1 |
| PCK2 |
| ENDOG |
| PPP1R12A |
| VSTM4 |
| ATP8B4 |
| KRT8 |
| RUNX1T1 |
| DENND2A |
| TRPC4 |
| TSPYL5 |
| CTSF |
| PTTG1 |
| MMP12 |
| UCHL3 |
| GGH |
| PSD |
| KRT18 |
| ZFP2 |
| SLC25A10 |
| MKI67 |
| TGFBR3 |
| IDH1 |
| CBLC |
| BACH2 |
| NME1 |
| EFS |
| E2F8 |
| TSTA3 |
| SEC23B |
| KLF11 |
| SLC22A18 |
| TSSC1 |
| GPI |
| ARID5B |
| C1orf21 |
| BLM |
| PBK |
| DPP3 |
| ASPM |
| CKAP2 |
| HK2 |
| TMEM35 |
| DTL |
| GMNN |
| CDCP1 |
| KIF20A |
| MIF |
| LRRC16A |
| LAMA4 |
| CLIC2 |
| TTK |
| SPINT2 |
| NCAPG |
| NUSAP1 |
| CCNB1 |
| LMNB2 |
| C16orf45 |
| KLF3-AS1 |
| F2RL1 |
| BCHE |
| RASIP1 |
| NCAM1 |
| CENPU |
| OSR2 |
| KIF14 |
| PAQR4 |
| TJP3 |
| SVIL |
| PPIEL |
| MELK |
| NDC80 |
| SLC37A1 |
| KIF23 |
| SLC39A4 |
| SPAG5 |
| MRPL13 |
| FZD5 |
| PRC1 |
| DBI |
| ITPKB |
| ARMCX1 |
| TBX3 |
| ESRP1 |
| PEG3 |
| HAND2-AS1 |
| LSR |
| CSTB |
| PGM5 |
| KIAA1462 |
| ST3GAL5 |
| BNC2 |
| CDIP1 |
| JAM3 |
| DIP2C |
| MXRA8 |
| KIF11 |
| RNF144A |
| CLDN7 |
| BHMT2 |
| WWC1 |
| PLS1 |
| ANK2 |
| CDC20 |
| PER3 |
| KDELR3 |
| EHBP1 |
| TRIP13 |
| CDC7 |
| CREB3L2 |
| TBL1X |
| CDO1 |
| UBE2C |
| CXCL12 |
| TOP2A |
| FEN1 |
| TUBG1 |
| KIF18A |
| ST14 |
| TMOD1 |
| GMDS |
| CTSZ |
| ARHGEF10 |
| NDC1 |
| CENPF |
| MAMLD1 |
| RNF38 |
| TPX2 |
| MITF |
| HOXD11 |
| LLGL2 |
| WT1-AS |
| APOBEC3B |
| AURKA |
| GABBR1 |
| FYN |
| MAP7 |
| RAD54B |
| USP18 |
| IRS2 |
| ZWINT |
| ENPP2 |
| MPDU1 |
| EPCAM |
| ECT2 |
| TMED3 |
| CENPE |
| KIAA1644 |
| TACC3 |
| WNT2 |
| CEP55 |
| CBX7 |
| SNRK |
| ST5 |
| DLGAP5 |
| RAD51AP1 |
| ENO1 |
| EPS8L2 |
| RPS6KA1 |
| ACACB |
| GAPDH |
| KIAA0101 |
| PGK1 |
| DHCR24 |
| VAMP8 |
| BIK |
| APEH |
| HDAC5 |
| MUC1 |
| ST8SIA1 |
| PCNA |
| PDS5B |
| S100A11 |
| TROAP |
| CMAHP |
| PSMD14 |
| TRPM4 |
| TPD52 |
| TK1 |
| KDR |
| GSPT2 |
| RBM47 |
| KIF15 |
| BUB1B |
| HJURP |
| NCALD |
| CDK5 |
| HMMR |
| SYT17 |
| SERINC1 |
| PDE8B |
| HOXD9 |
| DENND2D |
| BAX |
