# Supplementary material for: The modulatory properties of Astragalus membranaceus treatment on endometrial cancer: an integrated pharmacological method
Source: PeerJ. 2021 Aug 24;9:e11995. doi: 10.7717/peerj.11995 (PMC8395571; doi:10.7717/peerj.11995)
Supplement: Supplemental Information 2 [file peerj-09-11995-s002.docx]

**Table S2**: All compounds in *Astragalus membranaceus* and the targets

| Drug | MolId | MolName | Symbol |
| --- | --- | --- | --- |
| Huangqi | MOL000211 | Mairin | PGR |
| Huangqi | MOL000239 | Jaranol | NOS2 |
| Huangqi | MOL000239 | Jaranol | PTGS1 |
| Huangqi | MOL000239 | Jaranol | AR |
| Huangqi | MOL000239 | Jaranol | SCN5A |
| Huangqi | MOL000239 | Jaranol | PTGS2 |
| Huangqi | MOL000239 | Jaranol | ESR2 |
| Huangqi | MOL000239 | Jaranol | DPP4 |
| Huangqi | MOL000239 | Jaranol | HSP90AA1 |
| Huangqi | MOL000239 | Jaranol | CDK2 |
| Huangqi | MOL000239 | Jaranol | CHEK1 |
| Huangqi | MOL000239 | Jaranol | PRSS1 |
| Huangqi | MOL000239 | Jaranol | NCOA2 |
| Huangqi | MOL000239 | Jaranol | CALM1 |
| Huangqi | MOL000296 | hederagenin | PGR |
| Huangqi | MOL000296 | hederagenin | NCOA2 |
| Huangqi | MOL000296 | hederagenin | CHRM3 |
| Huangqi | MOL000296 | hederagenin | CHRM1 |
| Huangqi | MOL000296 | hederagenin | CHRM2 |
| Huangqi | MOL000296 | hederagenin | ADRA1B |
| Huangqi | MOL000296 | hederagenin | GABRA1 |
| Huangqi | MOL000296 | hederagenin | GRIA2 |
| Huangqi | MOL000296 | hederagenin | IGHG1 |
| Huangqi | MOL000296 | hederagenin | ADH1B |
| Huangqi | MOL000296 | hederagenin | ADH1C |
| Huangqi | MOL000296 | hederagenin | LYZD1 |
| Huangqi | MOL000296 | hederagenin | PTGS1 |
| Huangqi | MOL000296 | hederagenin | SCN5A |
| Huangqi | MOL000296 | hederagenin | PTGS2 |
| Huangqi | MOL000296 | hederagenin | RXRA |
| Huangqi | MOL000296 | hederagenin | SLC6A2 |
| Huangqi | MOL000033 | (3S,8S,9S,10R,13R,14S,17R)-10,13-dimethyl-17-[(2R,5S)-5-propan-2-yloctan-2-yl]-2,3,4,7,8,9,11,12,14,15,16,17-dodecahydro-1H-cyclopenta[a]phenanthren-3-ol | PGR |
| Huangqi | MOL000354 | isorhamnetin | NOS2 |
| Huangqi | MOL000354 | isorhamnetin | PTGS1 |
| Huangqi | MOL000354 | isorhamnetin | ESR1 |
| Huangqi | MOL000354 | isorhamnetin | AR |
| Huangqi | MOL000354 | isorhamnetin | PPARG |
| Huangqi | MOL000354 | isorhamnetin | PTGS2 |
| Huangqi | MOL000354 | isorhamnetin | ESR2 |
| Huangqi | MOL000354 | isorhamnetin | DPP4 |
| Huangqi | MOL000354 | isorhamnetin | MAPK14 |
| Huangqi | MOL000354 | isorhamnetin | GSK3B |
| Huangqi | MOL000354 | isorhamnetin | HSP90AA1 |
| Huangqi | MOL000354 | isorhamnetin | CDK2 |
| Huangqi | MOL000354 | isorhamnetin | PRSS1 |
| Huangqi | MOL000354 | isorhamnetin | CCNA2 |
| Huangqi | MOL000354 | isorhamnetin | NCOA2 |
| Huangqi | MOL000354 | isorhamnetin | CALM1 |
| Huangqi | MOL000354 | isorhamnetin | PYGM |
| Huangqi | MOL000354 | isorhamnetin | PPARD |
| Huangqi | MOL000354 | isorhamnetin | CHEK1 |
| Huangqi | MOL000354 | isorhamnetin | AKR1B1 |
| Huangqi | MOL000354 | isorhamnetin | NCOA1 |
| Huangqi | MOL000354 | isorhamnetin | F7 |
| Huangqi | MOL000354 | isorhamnetin | HTR |
| Huangqi | MOL000354 | isorhamnetin | ACHE |
| Huangqi | MOL000354 | isorhamnetin | GABRA1 |
| Huangqi | MOL000354 | isorhamnetin | MAOB |
| Huangqi | MOL000354 | isorhamnetin | GRIA2 |
| Huangqi | MOL000354 | isorhamnetin | RELA |
| Huangqi | MOL000354 | isorhamnetin | NCF1 |
| Huangqi | MOL000354 | isorhamnetin | OLR1 |
| Huangqi | MOL000371 | 3,9-di-O-methylnissolin | NOS2 |
| Huangqi | MOL000371 | 3,9-di-O-methylnissolin | PTGS1 |
| Huangqi | MOL000371 | 3,9-di-O-methylnissolin | CHRM3 |
| Huangqi | MOL000371 | 3,9-di-O-methylnissolin | HTR |
| Huangqi | MOL000371 | 3,9-di-O-methylnissolin | CHRM1 |
| Huangqi | MOL000371 | 3,9-di-O-methylnissolin | ESR1 |
| Huangqi | MOL000371 | 3,9-di-O-methylnissolin | ADRB1 |
| Huangqi | MOL000371 | 3,9-di-O-methylnissolin | SCN5A |
| Huangqi | MOL000371 | 3,9-di-O-methylnissolin | PTGS2 |
| Huangqi | MOL000371 | 3,9-di-O-methylnissolin | HTR3A |
| Huangqi | MOL000371 | 3,9-di-O-methylnissolin | ADRA2C |
| Huangqi | MOL000371 | 3,9-di-O-methylnissolin | RXRA |
| Huangqi | MOL000371 | 3,9-di-O-methylnissolin | ACHE |
| Huangqi | MOL000371 | 3,9-di-O-methylnissolin | ADRA1B |
| Huangqi | MOL000371 | 3,9-di-O-methylnissolin | ADRB2 |
| Huangqi | MOL000371 | 3,9-di-O-methylnissolin | ADRA1D |
| Huangqi | MOL000371 | 3,9-di-O-methylnissolin | OPRM1 |
| Huangqi | MOL000371 | 3,9-di-O-methylnissolin | GABRA1 |
| Huangqi | MOL000371 | 3,9-di-O-methylnissolin | PRSS1 |
| Huangqi | MOL000371 | 3,9-di-O-methylnissolin | NCOA2 |
| Huangqi | MOL000371 | 3,9-di-O-methylnissolin | CALM1 |
| Huangqi | MOL000378 | 7-O-methylisomucronulatol | NOS2 |
| Huangqi | MOL000378 | 7-O-methylisomucronulatol | PTGS1 |
| Huangqi | MOL000378 | 7-O-methylisomucronulatol | DRD1 |
| Huangqi | MOL000378 | 7-O-methylisomucronulatol | CHRM3 |
| Huangqi | MOL000378 | 7-O-methylisomucronulatol | HTR |
| Huangqi | MOL000378 | 7-O-methylisomucronulatol | KCNH2 |
| Huangqi | MOL000378 | 7-O-methylisomucronulatol | CHRM1 |
| Huangqi | MOL000378 | 7-O-methylisomucronulatol | ESR1 |
| Huangqi | MOL000378 | 7-O-methylisomucronulatol | AR |
| Huangqi | MOL000378 | 7-O-methylisomucronulatol | ADRB1 |
| Huangqi | MOL000378 | 7-O-methylisomucronulatol | SCN5A |
| Huangqi | MOL000378 | 7-O-methylisomucronulatol | PPARG |
| Huangqi | MOL000378 | 7-O-methylisomucronulatol | CHRM5 |
| Huangqi | MOL000378 | 7-O-methylisomucronulatol | PTGS2 |
| Huangqi | MOL000378 | 7-O-methylisomucronulatol | ADRA2C |
| Huangqi | MOL000378 | 7-O-methylisomucronulatol | CHRM4 |
| Huangqi | MOL000378 | 7-O-methylisomucronulatol | RXRA |
| Huangqi | MOL000378 | 7-O-methylisomucronulatol | OPRD1 |
| Huangqi | MOL000378 | 7-O-methylisomucronulatol | ADRA1A |
| Huangqi | MOL000378 | 7-O-methylisomucronulatol | CHRM2 |
| Huangqi | MOL000378 | 7-O-methylisomucronulatol | ADRA1B |
| Huangqi | MOL000378 | 7-O-methylisomucronulatol | SLC6A3 |
| Huangqi | MOL000378 | 7-O-methylisomucronulatol | ADRB2 |
| Huangqi | MOL000378 | 7-O-methylisomucronulatol | ADRA1D |
| Huangqi | MOL000378 | 7-O-methylisomucronulatol | SLC6A4 |
| Huangqi | MOL000378 | 7-O-methylisomucronulatol | ESR2 |
| Huangqi | MOL000378 | 7-O-methylisomucronulatol | GABRA1 |
| Huangqi | MOL000378 | 7-O-methylisomucronulatol | DPP4 |
| Huangqi | MOL000378 | 7-O-methylisomucronulatol | MAPK14 |
| Huangqi | MOL000378 | 7-O-methylisomucronulatol | GSK3B |
| Huangqi | MOL000378 | 7-O-methylisomucronulatol | HSP90AA1 |
| Huangqi | MOL000378 | 7-O-methylisomucronulatol | CDK2 |
| Huangqi | MOL000378 | 7-O-methylisomucronulatol | CHEK1 |
| Huangqi | MOL000378 | 7-O-methylisomucronulatol | RXRB |
| Huangqi | MOL000378 | 7-O-methylisomucronulatol | PRSS1 |
| Huangqi | MOL000378 | 7-O-methylisomucronulatol | CCNA2 |
| Huangqi | MOL000378 | 7-O-methylisomucronulatol | NCOA2 |
| Huangqi | MOL000378 | 7-O-methylisomucronulatol | CALM1 |
| Huangqi | MOL000379 | 9,10-dimethoxypterocarpan-3-O-β-D-glucoside | PTGS2 |
| Huangqi | MOL000379 | 9,10-dimethoxypterocarpan-3-O-β-D-glucoside | NCOA2 |
| Huangqi | MOL000380 | (6aR,11aR)-9,10-dimethoxy-6a,11a-dihydro-6H-benzofurano[3,2-c]chromen-3-ol | NOS2 |
| Huangqi | MOL000380 | (6aR,11aR)-9,10-dimethoxy-6a,11a-dihydro-6H-benzofurano[3,2-c]chromen-3-ol | PTGS1 |
| Huangqi | MOL000380 | (6aR,11aR)-9,10-dimethoxy-6a,11a-dihydro-6H-benzofurano[3,2-c]chromen-3-ol | CHRM3 |
| Huangqi | MOL000380 | (6aR,11aR)-9,10-dimethoxy-6a,11a-dihydro-6H-benzofurano[3,2-c]chromen-3-ol | HTR |
| Huangqi | MOL000380 | (6aR,11aR)-9,10-dimethoxy-6a,11a-dihydro-6H-benzofurano[3,2-c]chromen-3-ol | CHRM1 |
| Huangqi | MOL000380 | (6aR,11aR)-9,10-dimethoxy-6a,11a-dihydro-6H-benzofurano[3,2-c]chromen-3-ol | ESR1 |
| Huangqi | MOL000380 | (6aR,11aR)-9,10-dimethoxy-6a,11a-dihydro-6H-benzofurano[3,2-c]chromen-3-ol | SCN5A |
| Huangqi | MOL000380 | (6aR,11aR)-9,10-dimethoxy-6a,11a-dihydro-6H-benzofurano[3,2-c]chromen-3-ol | PTGS2 |
| Huangqi | MOL000380 | (6aR,11aR)-9,10-dimethoxy-6a,11a-dihydro-6H-benzofurano[3,2-c]chromen-3-ol | HTR3A |
| Huangqi | MOL000380 | (6aR,11aR)-9,10-dimethoxy-6a,11a-dihydro-6H-benzofurano[3,2-c]chromen-3-ol | RXRA |
| Huangqi | MOL000380 | (6aR,11aR)-9,10-dimethoxy-6a,11a-dihydro-6H-benzofurano[3,2-c]chromen-3-ol | ACHE |
| Huangqi | MOL000380 | (6aR,11aR)-9,10-dimethoxy-6a,11a-dihydro-6H-benzofurano[3,2-c]chromen-3-ol | ADRA1B |
| Huangqi | MOL000380 | (6aR,11aR)-9,10-dimethoxy-6a,11a-dihydro-6H-benzofurano[3,2-c]chromen-3-ol | ADRB2 |
| Huangqi | MOL000380 | (6aR,11aR)-9,10-dimethoxy-6a,11a-dihydro-6H-benzofurano[3,2-c]chromen-3-ol | ADRA1D |
| Huangqi | MOL000380 | (6aR,11aR)-9,10-dimethoxy-6a,11a-dihydro-6H-benzofurano[3,2-c]chromen-3-ol | GABRA1 |
| Huangqi | MOL000380 | (6aR,11aR)-9,10-dimethoxy-6a,11a-dihydro-6H-benzofurano[3,2-c]chromen-3-ol | HSP90AA1 |
| Huangqi | MOL000380 | (6aR,11aR)-9,10-dimethoxy-6a,11a-dihydro-6H-benzofurano[3,2-c]chromen-3-ol | PRSS1 |
| Huangqi | MOL000380 | (6aR,11aR)-9,10-dimethoxy-6a,11a-dihydro-6H-benzofurano[3,2-c]chromen-3-ol | NCOA2 |
| Huangqi | MOL000380 | (6aR,11aR)-9,10-dimethoxy-6a,11a-dihydro-6H-benzofurano[3,2-c]chromen-3-ol | NCOA1 |
| Huangqi | MOL000380 | (6aR,11aR)-9,10-dimethoxy-6a,11a-dihydro-6H-benzofurano[3,2-c]chromen-3-ol | CALM1 |
| Huangqi | MOL000380 | (6aR,11aR)-9,10-dimethoxy-6a,11a-dihydro-6H-benzofurano[3,2-c]chromen-3-ol | CHRM4 |
| Huangqi | MOL000387 | Bifendate | PTGS2 |
| Huangqi | MOL000387 | Bifendate | KDR |
| Huangqi | MOL000387 | Bifendate | MET |
| Huangqi | MOL000387 | Bifendate | HSP90AA1 |
| Huangqi | MOL000387 | Bifendate | PTGS1 |
| Huangqi | MOL000392 | formononetin | NOS2 |
| Huangqi | MOL000392 | formononetin | PTGS1 |
| Huangqi | MOL000392 | formononetin | CHRM1 |
| Huangqi | MOL000392 | formononetin | ESR1 |
| Huangqi | MOL000392 | formononetin | AR |
| Huangqi | MOL000392 | formononetin | PPARG |
| Huangqi | MOL000392 | formononetin | PTGS2 |
| Huangqi | MOL000392 | formononetin | RXRA |
| Huangqi | MOL000392 | formononetin | ADRA1A |
| Huangqi | MOL000392 | formononetin | SLC6A3 |
| Huangqi | MOL000392 | formononetin | ADRB2 |
| Huangqi | MOL000392 | formononetin | SLC6A4 |
| Huangqi | MOL000392 | formononetin | ESR2 |
| Huangqi | MOL000392 | formononetin | DPP4 |
| Huangqi | MOL000392 | formononetin | MAPK14 |
| Huangqi | MOL000392 | formononetin | GSK3B |
| Huangqi | MOL000392 | formononetin | HSP90AA1 |
| Huangqi | MOL000392 | formononetin | CDK2 |
| Huangqi | MOL000392 | formononetin | MAOB |
| Huangqi | MOL000392 | formononetin | CHEK1 |
| Huangqi | MOL000392 | formononetin | PRSS1 |
| Huangqi | MOL000392 | formononetin | CCNA2 |
| Huangqi | MOL000392 | formononetin | CALM1 |
| Huangqi | MOL000392 | formononetin | PKIA |
| Huangqi | MOL000392 | formononetin | HTR |
| Huangqi | MOL000392 | formononetin | ACHE |
| Huangqi | MOL000392 | formononetin | JUN |
| Huangqi | MOL000392 | formononetin | PPARG |
| Huangqi | MOL000392 | formononetin | IL4 |
| Huangqi | MOL000392 | formononetin | ATP5F1B |
| Huangqi | MOL000392 | formononetin | ND6 |
| Huangqi | MOL000392 | formononetin | HSD3B2 |
| Huangqi | MOL000392 | formononetin | HSD3B1 |
| Huangqi | MOL000417 | Calycosin | NOS2 |
| Huangqi | MOL000417 | Calycosin | PTGS1 |
| Huangqi | MOL000417 | Calycosin | ESR1 |
| Huangqi | MOL000417 | Calycosin | AR |
| Huangqi | MOL000417 | Calycosin | PPARG |
| Huangqi | MOL000417 | Calycosin | PTGS2 |
| Huangqi | MOL000417 | Calycosin | RXRA |
| Huangqi | MOL000417 | Calycosin | ESR2 |
| Huangqi | MOL000417 | Calycosin | DPP4 |
| Huangqi | MOL000417 | Calycosin | MAPK14 |
| Huangqi | MOL000417 | Calycosin | GSK3B |
| Huangqi | MOL000417 | Calycosin | HSP90AA1 |
| Huangqi | MOL000417 | Calycosin | CDK2 |
| Huangqi | MOL000417 | Calycosin | CHEK1 |
| Huangqi | MOL000417 | Calycosin | PRSS1 |
| Huangqi | MOL000417 | Calycosin | CCNA2 |
| Huangqi | MOL000417 | Calycosin | NCOA2 |
| Huangqi | MOL000417 | Calycosin | CALM1 |
| Huangqi | MOL000417 | Calycosin | ADRB2 |
| Huangqi | MOL000422 | kaempferol | NOS2 |
| Huangqi | MOL000422 | kaempferol | PTGS1 |
| Huangqi | MOL000422 | kaempferol | AR |
| Huangqi | MOL000422 | kaempferol | PPARG |
| Huangqi | MOL000422 | kaempferol | PTGS2 |
| Huangqi | MOL000422 | kaempferol | HSP90AA1 |
| Huangqi | MOL000422 | kaempferol | NCOA2 |
| Huangqi | MOL000422 | kaempferol | DPP4 |
| Huangqi | MOL000422 | kaempferol | PRSS1 |
| Huangqi | MOL000422 | kaempferol | PGR |
| Huangqi | MOL000422 | kaempferol | HTR |
| Huangqi | MOL000422 | kaempferol | CHRM1 |
| Huangqi | MOL000422 | kaempferol | ACHE |
| Huangqi | MOL000422 | kaempferol | SLC6A2 |
| Huangqi | MOL000422 | kaempferol | CHRM2 |
| Huangqi | MOL000422 | kaempferol | ADRA1B |
| Huangqi | MOL000422 | kaempferol | GABRA1 |
| Huangqi | MOL000422 | kaempferol | F7 |
| Huangqi | MOL000422 | kaempferol | CALM1 |
| Huangqi | MOL000422 | kaempferol | RELA |
| Huangqi | MOL000422 | kaempferol | IKBKB |
| Huangqi | MOL000422 | kaempferol | AKT1 |
| Huangqi | MOL000422 | kaempferol | BCL2 |
| Huangqi | MOL000422 | kaempferol | BAX |
| Huangqi | MOL000422 | kaempferol | TNFSF15 |
| Huangqi | MOL000422 | kaempferol | JUN |
| Huangqi | MOL000422 | kaempferol | AHSA1 |
| Huangqi | MOL000422 | kaempferol | CASP3 |
| Huangqi | MOL000422 | kaempferol | MAPK8 |
| Huangqi | MOL000422 | kaempferol | MMP1 |
| Huangqi | MOL000422 | kaempferol | STAT1 |
| Huangqi | MOL000422 | kaempferol | CDK1 |
| Huangqi | MOL000422 | kaempferol | PPARG |
| Huangqi | MOL000422 | kaempferol | HMOX1 |
| Huangqi | MOL000422 | kaempferol | CYP3A4 |
| Huangqi | MOL000422 | kaempferol | CYP1A2 |
| Huangqi | MOL000422 | kaempferol | CYP1A1 |
| Huangqi | MOL000422 | kaempferol | ICAM1 |
| Huangqi | MOL000422 | kaempferol | SELE |
| Huangqi | MOL000422 | kaempferol | VCAM1 |
| Huangqi | MOL000422 | kaempferol | NR1I2 |
| Huangqi | MOL000422 | kaempferol | CYP1B1 |
| Huangqi | MOL000422 | kaempferol | ALOX5 |
| Huangqi | MOL000422 | kaempferol | HAS2 |
| Huangqi | MOL000422 | kaempferol | GSTP1 |
| Huangqi | MOL000422 | kaempferol | AHR |
| Huangqi | MOL000422 | kaempferol | PSMD3 |
| Huangqi | MOL000422 | kaempferol | SLC2A4 |
| Huangqi | MOL000422 | kaempferol | NR1I3 |
| Huangqi | MOL000422 | kaempferol | INSRR |
| Huangqi | MOL000422 | kaempferol | DIO1 |
| Huangqi | MOL000422 | kaempferol | PPP3CA |
| Huangqi | MOL000422 | kaempferol | GSTM1 |
| Huangqi | MOL000422 | kaempferol | GSTM2 |
| Huangqi | MOL000422 | kaempferol | AKR1C3 |
| Huangqi | MOL000422 | kaempferol | SLPI |
| Huangqi | MOL000433 | FA | CDK2 |
| Huangqi | MOL000433 | FA | HTR |
| Huangqi | MOL000433 | FA | GSK3B |
| Huangqi | MOL000442 | 1,7-Dihydroxy-3,9-dimethoxy pterocarpene | PTGS2 |
| Huangqi | MOL000442 | 1,7-Dihydroxy-3,9-dimethoxy pterocarpene | RXRA |
| Huangqi | MOL000442 | 1,7-Dihydroxy-3,9-dimethoxy pterocarpene | HSP90AA1 |
| Huangqi | MOL000442 | 1,7-Dihydroxy-3,9-dimethoxy pterocarpene | PRSS1 |
| Huangqi | MOL000098 | quercetin | PTGS1 |
| Huangqi | MOL000098 | quercetin | AR |
| Huangqi | MOL000098 | quercetin | PPARG |
| Huangqi | MOL000098 | quercetin | PTGS2 |
| Huangqi | MOL000098 | quercetin | HSP90AA1 |
| Huangqi | MOL000098 | quercetin | NCOA2 |
| Huangqi | MOL000098 | quercetin | DPP4 |
| Huangqi | MOL000098 | quercetin | AKR1B1 |
| Huangqi | MOL000098 | quercetin | PRSS1 |
| Huangqi | MOL000098 | quercetin | HTR |
| Huangqi | MOL000098 | quercetin | KCNH2 |
| Huangqi | MOL000098 | quercetin | SCN5A |
| Huangqi | MOL000098 | quercetin | ADRB2 |
| Huangqi | MOL000098 | quercetin | MMP3 |
| Huangqi | MOL000098 | quercetin | F7 |
| Huangqi | MOL000098 | quercetin | RXRA |
| Huangqi | MOL000098 | quercetin | ACHE |
| Huangqi | MOL000098 | quercetin | GABRA1 |
| Huangqi | MOL000098 | quercetin | MAOB |
| Huangqi | MOL000098 | quercetin | RELA |
| Huangqi | MOL000098 | quercetin | EGFR |
| Huangqi | MOL000098 | quercetin | AKT1 |
| Huangqi | MOL000098 | quercetin | VEGFA |
| Huangqi | MOL000098 | quercetin | CCND1 |
| Huangqi | MOL000098 | quercetin | BCL2 |
| Huangqi | MOL000098 | quercetin | BCL2L1 |
| Huangqi | MOL000098 | quercetin | FOS |
| Huangqi | MOL000098 | quercetin | CDKN1A |
| Huangqi | MOL000098 | quercetin | EIF6 |
| Huangqi | MOL000098 | quercetin | BAX |
| Huangqi | MOL000098 | quercetin | CASP9 |
| Huangqi | MOL000098 | quercetin | PLAU |
| Huangqi | MOL000098 | quercetin | MMP2 |
| Huangqi | MOL000098 | quercetin | MMP9 |
| Huangqi | MOL000098 | quercetin | MAPK1 |
| Huangqi | MOL000098 | quercetin | IL10RB |
| Huangqi | MOL000098 | quercetin | EGF |
| Huangqi | MOL000098 | quercetin | RB1 |
| Huangqi | MOL000098 | quercetin | TNFSF15 |
| Huangqi | MOL000098 | quercetin | JUN |
| Huangqi | MOL000098 | quercetin | IL6 |
| Huangqi | MOL000098 | quercetin | AHSA1 |
| Huangqi | MOL000098 | quercetin | CASP3 |
| Huangqi | MOL000098 | quercetin | TP63 |
| Huangqi | MOL000098 | quercetin | ELK1 |
| Huangqi | MOL000098 | quercetin | NFKBIA |
| Huangqi | MOL000098 | quercetin | POR |
| Huangqi | MOL000098 | quercetin | ODC1 |
| Huangqi | MOL000098 | quercetin | CASP8 |
| Huangqi | MOL000098 | quercetin | TOP1 |
| Huangqi | MOL000098 | quercetin | RAF1 |
| Huangqi | MOL000098 | quercetin | SOD1 |
| Huangqi | MOL000098 | quercetin | PRKCA |
| Huangqi | MOL000098 | quercetin | MMP1 |
| Huangqi | MOL000098 | quercetin | HIF1A |
| Huangqi | MOL000098 | quercetin | STAT1 |
| Huangqi | MOL000098 | quercetin | RUNX1T1 |
| Huangqi | MOL000098 | quercetin | CDK1 |
| Huangqi | MOL000098 | quercetin | HSPA5 |
| Huangqi | MOL000098 | quercetin | ERBB2 |
| Huangqi | MOL000098 | quercetin | PPARG |
| Huangqi | MOL000098 | quercetin | ACACA |
| Huangqi | MOL000098 | quercetin | HMOX1 |
| Huangqi | MOL000098 | quercetin | CYP3A4 |
| Huangqi | MOL000098 | quercetin | CYP1A2 |
| Huangqi | MOL000098 | quercetin | CAV1 |
| Huangqi | MOL000098 | quercetin | MYC |
| Huangqi | MOL000098 | quercetin | F3 |
| Huangqi | MOL000098 | quercetin | GJA1 |
| Huangqi | MOL000098 | quercetin | CYP1A1 |
| Huangqi | MOL000098 | quercetin | ICAM1 |
| Huangqi | MOL000098 | quercetin | IL1B |
| Huangqi | MOL000098 | quercetin | CCL2 |
| Huangqi | MOL000098 | quercetin | SELE |
| Huangqi | MOL000098 | quercetin | VCAM1 |
| Huangqi | MOL000098 | quercetin | PTGER3 |
| Huangqi | MOL000098 | quercetin | IL8RA |
| Huangqi | MOL000098 | quercetin | PRKCB |
| Huangqi | MOL000098 | quercetin | BIRC5 |
| Huangqi | MOL000098 | quercetin | DUOX2 |
| Huangqi | MOL000098 | quercetin | NOS3 |
| Huangqi | MOL000098 | quercetin | HSPB1 |
| Huangqi | MOL000098 | quercetin | SULT1E1 |
| Huangqi | MOL000098 | quercetin | MGAM |
| Huangqi | MOL000098 | quercetin | IL2 |
| Huangqi | MOL000098 | quercetin | NR1I2 |
| Huangqi | MOL000098 | quercetin | CYP1B1 |
| Huangqi | MOL000098 | quercetin | CCNB1 |
| Huangqi | MOL000098 | quercetin | PLAT |
| Huangqi | MOL000098 | quercetin | THBD |
| Huangqi | MOL000098 | quercetin | SERPINE1 |
| Huangqi | MOL000098 | quercetin | COL1A1 |
| Huangqi | MOL000098 | quercetin | IFNGR1 |
| Huangqi | MOL000098 | quercetin | ALOX5 |
| Huangqi | MOL000098 | quercetin | IL1A |
| Huangqi | MOL000098 | quercetin | MPO |
| Huangqi | MOL000098 | quercetin | TOP2A |
| Huangqi | MOL000098 | quercetin | NCF1 |
| Huangqi | MOL000098 | quercetin | ABCG2 |
| Huangqi | MOL000098 | quercetin | HAS2 |
| Huangqi | MOL000098 | quercetin | GSTP1 |
| Huangqi | MOL000098 | quercetin | NFE2L2 |
| Huangqi | MOL000098 | quercetin | NQO1 |
| Huangqi | MOL000098 | quercetin | PARP1 |
| Huangqi | MOL000098 | quercetin | AHR |
| Huangqi | MOL000098 | quercetin | PSMD3 |
| Huangqi | MOL000098 | quercetin | SLC2A4 |
| Huangqi | MOL000098 | quercetin | COL3A1 |
| Huangqi | MOL000098 | quercetin | CXCL11 |
| Huangqi | MOL000098 | quercetin | CXCL2 |
| Huangqi | MOL000098 | quercetin | DCAF5 |
| Huangqi | MOL000098 | quercetin | NR1I3 |
| Huangqi | MOL000098 | quercetin | CHEK2 |
| Huangqi | MOL000098 | quercetin | INSRR |
| Huangqi | MOL000098 | quercetin | CLDN4 |
| Huangqi | MOL000098 | quercetin | PPARA |
| Huangqi | MOL000098 | quercetin | PPARD |
| Huangqi | MOL000098 | quercetin | HSF1 |
| Huangqi | MOL000098 | quercetin | CRP |
| Huangqi | MOL000098 | quercetin | CXCL10 |
| Huangqi | MOL000098 | quercetin | CHUK |
| Huangqi | MOL000098 | quercetin | SPP1 |
| Huangqi | MOL000098 | quercetin | RUNX2 |
| Huangqi | MOL000098 | quercetin | RASSF1 |
| Huangqi | MOL000098 | quercetin | E2F1 |
| Huangqi | MOL000098 | quercetin | E2F2 |
| Huangqi | MOL000098 | quercetin | ACPP |
| Huangqi | MOL000098 | quercetin | CTSD |
| Huangqi | MOL000098 | quercetin | IGFBP3 |
| Huangqi | MOL000098 | quercetin | IGF2 |
| Huangqi | MOL000098 | quercetin | CD40LG |
| Huangqi | MOL000098 | quercetin | IRF1 |
| Huangqi | MOL000098 | quercetin | ERBB3 |
| Huangqi | MOL000098 | quercetin | PON1 |
| Huangqi | MOL000098 | quercetin | DIO1 |
| Huangqi | MOL000098 | quercetin | PCOLCE |
| Huangqi | MOL000098 | quercetin | NPEPPS |
| Huangqi | MOL000098 | quercetin | HK2 |
| Huangqi | MOL000098 | quercetin | RASA1 |
| Huangqi | MOL000098 | quercetin | GSTM1 |
| Huangqi | MOL000098 | quercetin | GSTM2 |
